# Supplementary material for: Viscosity jump in the lower mantle inferred from melting curves of ferropericlase
Source: Nat Commun. 2017 Dec 8;8:1997. doi: 10.1038/s41467-017-02263-z (PMC5722891; doi:10.1038/s41467-017-02263-z)
Supplement: Supplementary file 1 — Supplementary Information [file 41467_2017_2263_MOESM1_ESM.pdf]

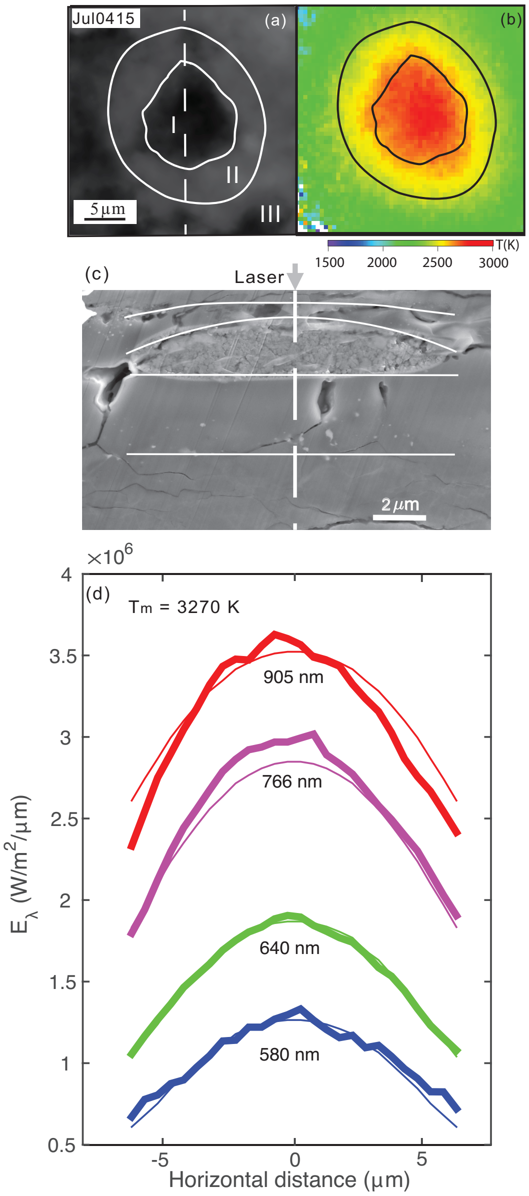

Supplementary Figure 1. Illustration of temperature calculation and correction methods. (a) Optical image; Regions I, II, and III are molten area, coexisting solid, and starting material, respectively. Dashed lines in optical images are the cross section position. **(b)** Two-dimensional temperature map. **(c)** FIB'd cross section of heated area corresponding to the vertical dashed line in (a). **(d)** Corresponding inverse modeling results. The thick lines are the horizontal intensities of radiation detected, and the thin lines are the fitted results with the corresponding wavelength labeled. Figure is modified from ref. 1.

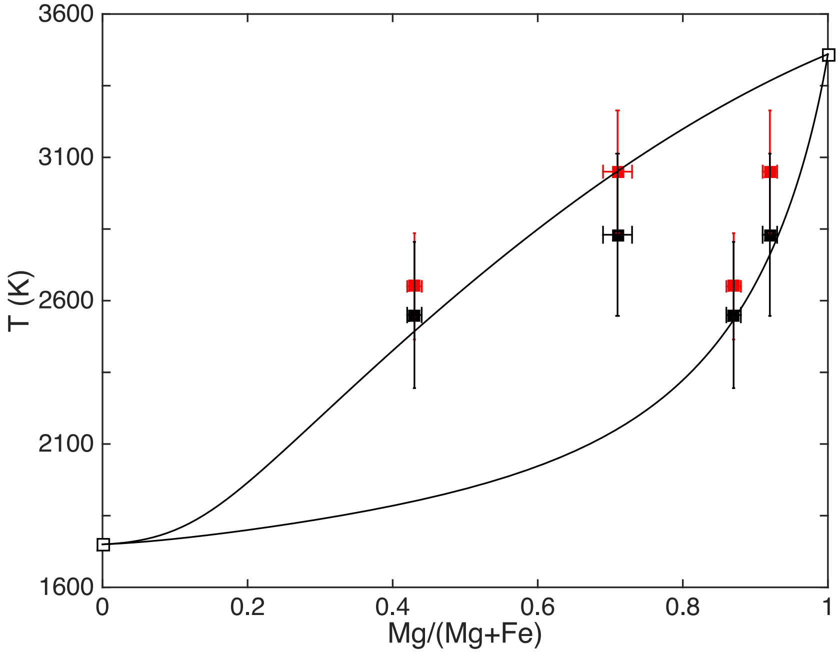

Supplementary Figure 2. The phase diagram of ferropericlase at 3 GPa calculated based on the symmetric regular solution model. Both the original data[2] (red) and after temperature correction[1] (black) are shown for comparison. The input parameters for the regular solution model are $T_{m,FeO}$ = 1750 K, $T_{m,MgO}$ = 3460 K, $\Delta H_{m,FeO}$ = 36 kJ mol^{-1}, $\Delta H_{m,MgO}$ = 87 kJ mol^{-1}, $W^{solid}_{FeO\text{-}MgO}$ = 11 kJ mol^{-1}, $W^{liquid}_{FeO\text{-}MgO}$ = -30 kJ mol^{-1}.

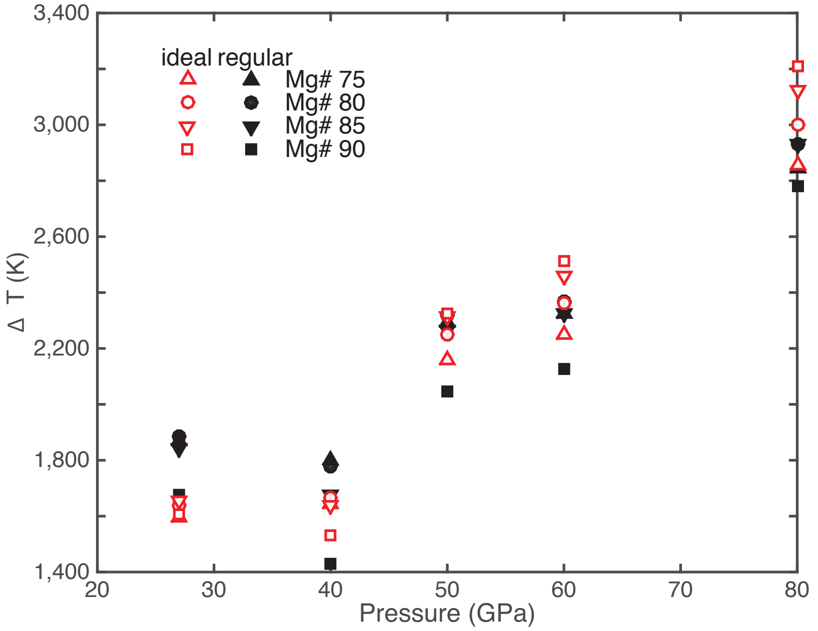

Supplementary Figure 3. The difference of solidus temperatures obtained by linear extrapolation from melting temperatures of the pure end-members to those obtained in this study. Red, open symbols represent ideal solution model and black, solid symbols represent the regular solution model.

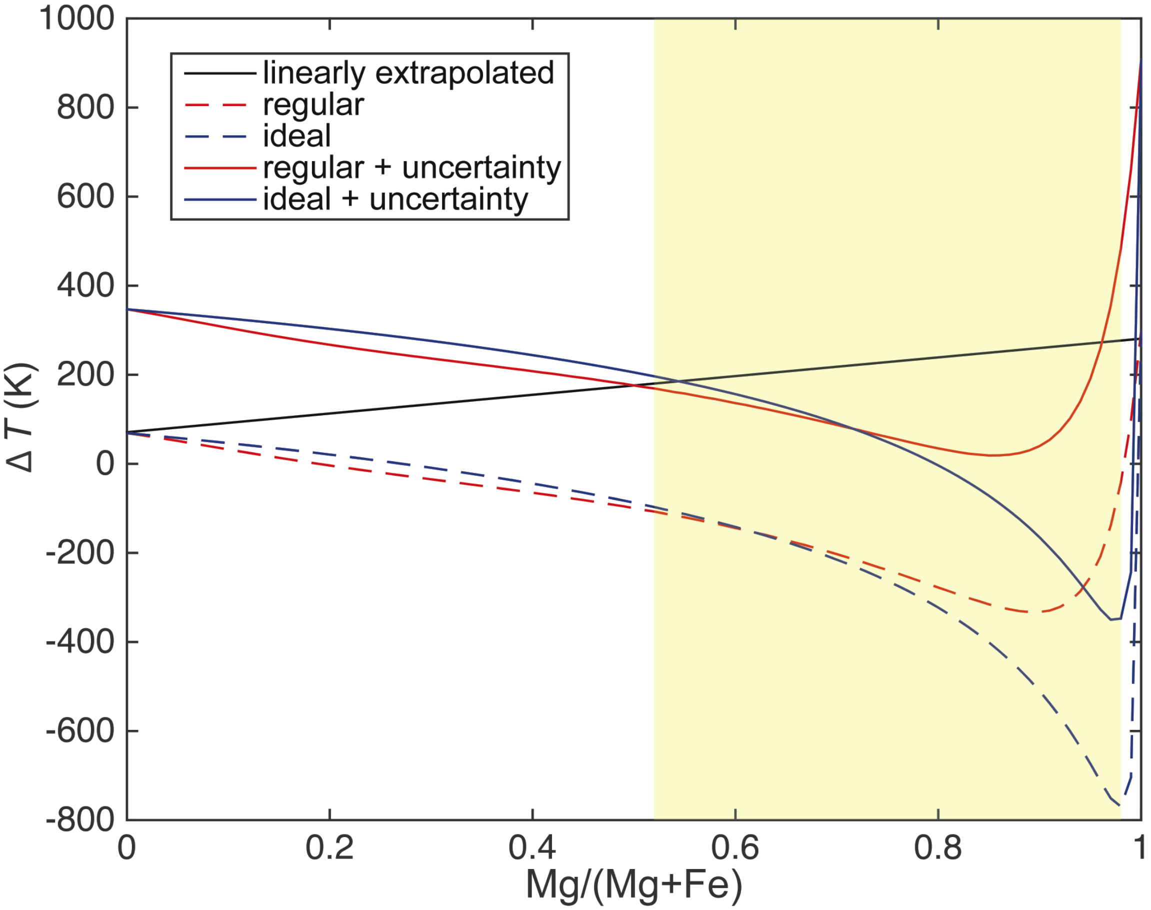

Supplementary Figure 4. Linearly extrapolated temperature increments compared with temperature increments determined from phase diagrams from 40 to 50 GPa. Comparison between the linearly extrapolated temperature increments from 40 to 50 GPa (black, solid curve) and those from the phase diagram calculation based on both the regular solution model and the ideal solution model with (solid curves) and without (dashed curves) including the 10% uncertainty in the solidus melting temperature at 50 GPa. The shaded region highlights the region where extra solidus temperature depression at 50 GPa occurs likely due to the spin crossover.

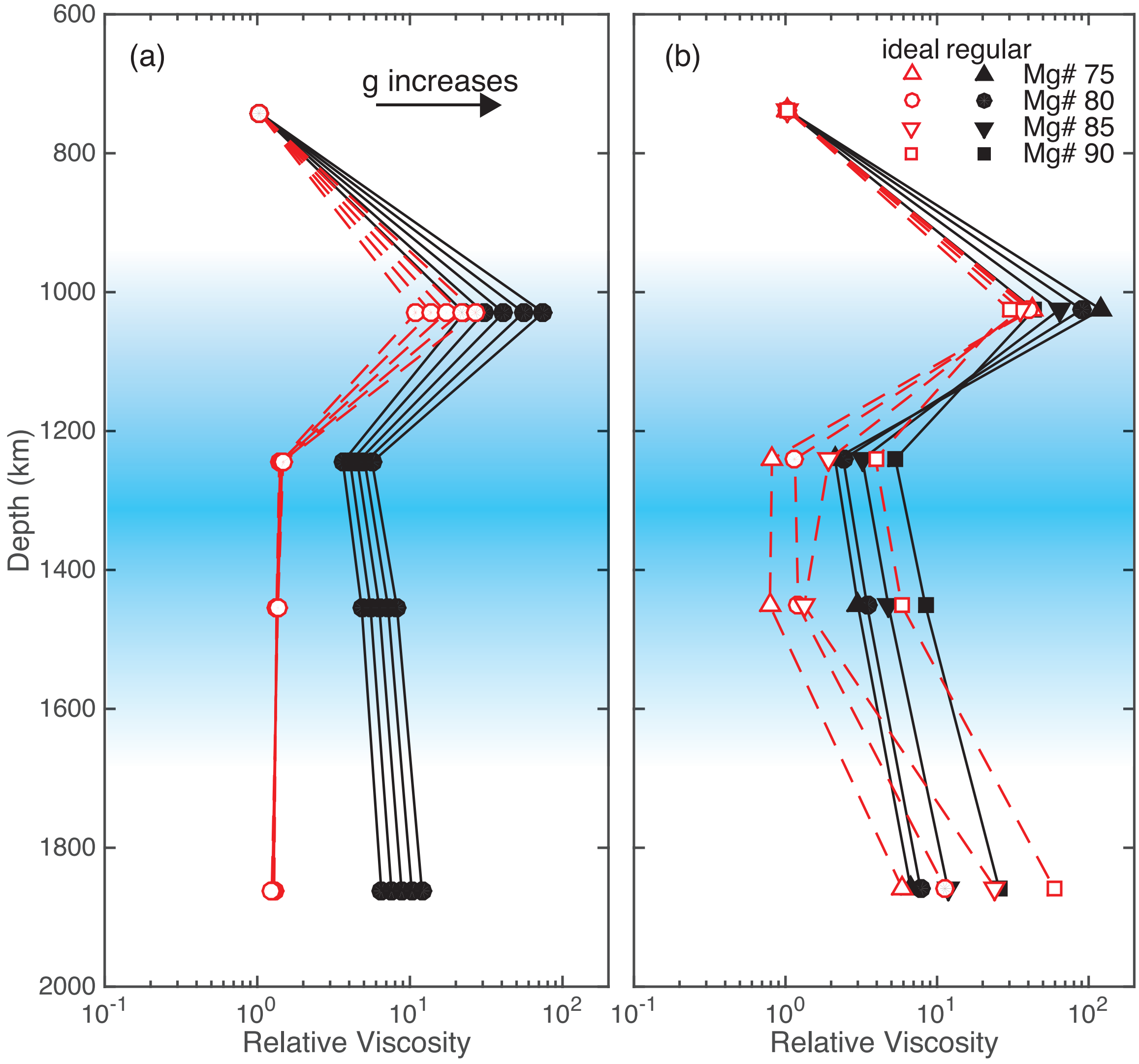

Supplementary Figure 5. The relative radial viscosity profiles of ferropericlase inferred from homologous temperature scaling. (a) relative viscosity profiles calculated based on the solidus melting curve of $(Mg_{0.80}Fe_{0.20})O$ for a range of plausible *g* values (i.e., g = 10-14). **(b)** relative viscosity profiles calculated based on the liquidus of ferropericlase of different Mg# assuming g = 12. Red open (black solid) markers represent the relative viscosity calculated using the melting temperatures assuming ideal (regular) solution model. Uncertainties on the viscosity are approximately enveloped by the values given by the solidus (Fig. 4) and liquidus, and two different solid solution models under two different geotherms. The blue shaded regions include spin transition pressure range for Mg#75-90 at 300 K (~35-70 GPa)[3]. The spin transition pressure ranges at corresponding high temperatures are likely broader but begin at pressures similar to those at 300 K[4,5]. Curves are drawn as a guide for the eye.

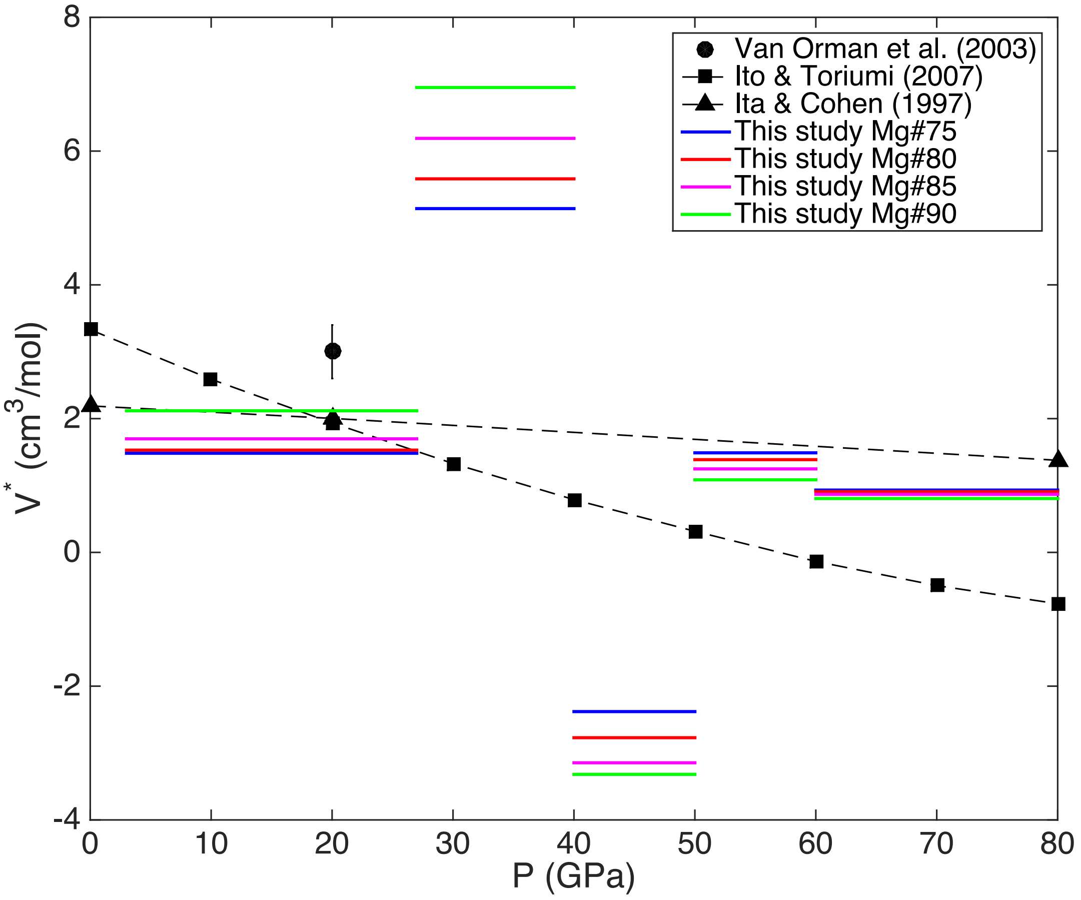

Supplementary Figure 6. Activation volume of self diffusion of Mg in (Mg,Fe)O ferropericlase. Here g = 12 and Mg# = 75 (blue), 80 (red), 85 (pink) and 90 (green) compared with activation volume of self diffusion of Mg in pure MgO obtained in previous studies[6,7,8].

Supplementary Table 1. Summary of the experimental conditions and results. Compositions are measured for the quenched samples after laser heating and the standard deviations of multiple measurements at each region are shown in parentheses. Uncertainties in pressure (95% confidence level) are given in parentheses. Temperature uncertainties are ~7% for perimeter and hottest point values and is ~10% for inverse modeling (95% confidence level)[1]. Temperatures used in the phase diagrams are those computed by the inverse modeling.

Run#	P (GPa)	Starting Material (Mg#)	Melt (Mg#)	Coexisting Solid (Mg#)	Temperature (K)		
					Perimeter T_a	Hottest point T_a	Inverse modeling T_m
13-0814 [a, b]	27(1)	91(1)	73(1)	94(1)	4300	4655	3790
140610 [b]	27(1)	91(1)	68(1)	95(1)	3650	3840	3450
Fp2002B [a]	27(1)	20(1)	59(1)	87(1)	3000	3204	3100
Fp200140G_D	40(1)	20(1)	36(1)	62(1)	2600	3047	3150
Fp200140G_B	40(1)	20(1)	37(3)	69(1)	2700	3056	3200
14-0506_45G [b]	40(1)	90(1)	67(3)	93(1)	5000	5505	4400
14-0426 [b]	40(1)	90(1)	55(2)	92(1)	4300	4517	3910
May2715C [a, c]	51(1)	88(1)	71(1)	91(1)	4250	4500	3800
Jan311650GC [a]	50(1)	81(1)	51(3)	84(1)	3000	3209	3200
Fp2006C [d]	60(1)	20(1)	10(5)	75(4)	2700	3076	2990
Jul0415 [c]	60(1)	81(1)	59(1)	90(1)	2600	2750	3270
Jun1115A	60(1)	88(1)	71(2)	95(1)	4100	4355	4070
Fp800180GA [e]	80(1)	82(1)	62(1)	91(1)	-	-	4150
Fp800180GB [f]	80(1)	82(1)	50(5)	88(2)	3200	3455	3550
Fp201283G [g]	83(1)	23(1)	<9	76(1)	2800	3405	3450

[a]Intensity data of thermal radiation saturated in one wavelength. Three-color temperature fit was implemented, see ref. 1 for more details.

[b]Data taken from ref. 2. Note the temperatures are re-calculated using mutli-layer inverse modeling, which show a large contrast with the temperatures used in the ref. 2.

[c]Data taken from ref. 1.

[d]Data taken from The melt composition is from semi-quantitative analysis by Energy Dispersive Spectroscopy (EDS).

[e]Intensity data of thermal radiation saturated in all four wavelengths. Temperature calculated by the power ratio used for Fp800180GA and Fp800180GB and the inverse modeling T_m of Fp800180GB.

[f]The chemical composition of the melt is estimated from the degree of melting which is determined by the areas of coexisting solid and melt from the optical images.

[g]The melt is too small (< 1 μm) to measure precisely using WDS. In order to get a estimate of the composition, we compare the melt and $(Mg_{0.09}Fe_{0.81})O$ under BSE. The melt tends to be "brighter" than $(Mg_{0.09}Fe_{0.81})O$, which indicates that it is more iron-rich than $(Mg_{0.09}Fe_{0.81})O$.

Mg# = 100×Mg/(Mg+Fe)

Supplementary Table 2. Best-fitting thermodynamic parameters using the ideal solution model. Uncertainties in pressure and fitted parameters at each region are shown in parentheses.

Pressure (GPa)	27(1)	40 (1)	50(1)	60(1)	80(1)
ΔH_{MgO} (kJ mol^{-1})	31(10)	43(14)	25(7)	26(10)	32(9)
ΔH_{FeO} (kJ mol^{-1})	105(30)	100(40)	160(56)	244(35)	280(43)

Supplementary Table 3. Best fitting thermodynamic parameters using the regular solution model. Uncertainties in pressure and fitted parameters at each region are shown in parentheses.

Pressure (GPa)	27(1)	40 (1)	50(1)	60(1)	80(1)
ΔH_{MgO} (kJ mol^{-1})	33(11)	55(15)	23(7)	24(9)	24(7)
ΔH_{FeO} (kJ mol^{-1})	47(13)	49(14)	49(21)	58(25)	75(18)
$W^{liquid}_{FeO\text{-}MgO}$ (kJ mol^{-1})	-16(4)	-10(8)	10(5)	12(5)	23(6)
$W^{solid}_{FeO\text{-}MgO}$ (kJ mol^{-1})	18(6)	25(6)	32(5)	35(6)	39(7)

Supplementary Note 1: Attainment of chemical equilibrium

(Mg,Fe)O melt is thought to be homogenized instantaneously due to the fast diffusion rate of elements[2, 9]. In contrast, the Mg-Fe interdiffusivities in (Mg,Fe)O solid are relatively sluggish and are strongly dependent on temperature, pressure and chemical compositions[10, 11, 12]. We calculated the length scales corresponding to our experiment durations using the experimentally determined chemical diffusion relation. The length scales are overall larger than the maximum dimensions of the coexisting solid observed (~1-4 μm). Therefore, the chemical equilibrium at least between melt and coexisting solid is achieved during the experiment. Following ref. 2, we also measured the chemical composition within the melts and coexisting solids and found the standard deviation is generally smaller than 3% as indicated in the Supplementary Table 1.

As noted above, we use pure MgO as an insulation layer for iron-rich starting materials. Consequently, the reaction between the starting materials and the MgO insulation layer are inevitable due to the large chemical gradient. But chemical equilibrium between the melt and coexisting solid is not affected. As argued above, what determines the chemical equilibrium is the Fe-Mg inter-diffusion rate. And both the inter-diffusion rate and chemical characterization confirm that chemical homogenization occurs within the length scale of melt and coexisting solids in our experiments. The only effect is that the starting materials are diluted and the melt is more Fe depleted than the initial starting materials for samples Fp2002B, Fp200140G_D and Fp200140G_B.

Supplementary Note 2: Comparison with the low pressure melting data of ferropericlase

Both Zhang and Fei[13] and Du and Lee[2] conducted (Mg,Fe)O melting experiments at pressures lower than 27 GPa. However, the results are in sharp contrast with each other, with ref. 13 being around several hundreds of K higher. As pointed out by Du and Lee [2], this discrepancy is likely due to the indirect temperature measurements beyond the limit of thermocouples (typically for temperatures > 2300 K) and therefore the extrapolation of a linear temperature and power relationship for the heater may be an invalid assumption at the high temperatures like those reported in ref. 13. In addition, both inferred FeO and MgO melting temperatures by ref. 13 at evaluated pressures are several hundreds of K higher than the direct experiment measurements[14] and many other first principle computations[9, 15, 16]. As such, we do not include the melting data by ref. 13 for comparison.

In order to compare our results with the low pressure melting experiments by Du and Lee[2], extrapolation and interpolation of thermodynamic parameters are necessary. As discussed above, the regular solution model might be more appropriate to describe the MgO-FeO system in the pressure range we examined. We therefore use the regular solution model to calculate the phase diagram at low pressures in case the ideal solution model is insufficient to describe the mixing of MgO and FeO. As described above, six parameters ($T_{\mathrm{m,FeO}}$, $T_{\mathrm{m,MgO}}$, $\Delta H_{\mathrm{m,FeO}}$, $\Delta H_{\mathrm{m,MgO}}$, $W^{\mathrm{solid}}_{\mathrm{FeO\text{-}MgO}}$, $W^{\mathrm{liquid}}_{\mathrm{FeO\text{-}MgO}}$) are needed to construct a phase diagram using the symmetric regular solution model. $T_{\mathrm{m,FeO}}$ and $T_{\mathrm{m,MgO}}$ are taken from previous studies following our convention. Both thermodynamic models[17, 18] suggest the same $\Delta H_{\mathrm{m,FeO}}$ of 36 kJ mol^{-1} at 3 GPa. Following our convention $W^{\mathrm{solid}}_{\mathrm{FeO\text{-}MgO}}$ is set as fitting

parameters with bounds 4.4–11.4 kJ mol^{-1} defined by the equations (3) and (4) of the main text. The remaining question is how to constrain the $\Delta H_{\text{m,MgO}}$ and $W^{\text{liquid}}_{\text{FeO-MgO}}$ at pressures less than 27 GPa based on our fitted values at high pressures and available literature data. For $W^{\text{liquid}}_{\text{FeO-MgO}}$, we follow refs.17, 19 and fit $W^{\text{liquid}}_{\text{FeO-MgO}}$ as a linear function of pressure,

$$W^{\text{liquid}}_{\text{FeO-MgO}}(\text{kJ/mol}) = -35.92(\pm 6.49) + 0.78(\pm 0.12)P \quad (11)$$

Experimental data of $\Delta H_{\text{m,MgO}}$ is only available at 1 bar (87 kJ mol^{-1})[20]. As discussed above, the reported $\Delta H_{\text{m,MgO}}$ values at elevated pressures show large discrepancies. But interestingly, most computations yield very similar melting curves of MgO, which indicates that quantification of entropy/volume of melting for MgO is difficult and controversial in first-principles computations. Specifically, the difficulties may come from the size of ensembles, effects of the surface energy and so on (see more discussion in refs. 9, 15). Our inferred $\Delta H_{\text{m,MgO}}$ values slightly decrease with pressures from 27 GPa to 50 GPa and then remain roughly constant upon increasing pressures with $\Delta H_{\text{m,MgO}}$ at 40 GPa being exceptionally higher. Nevertheless, this trend is generally consistent with ref. 20 . To avoid the large influence of uncertainty of $\Delta H_{\text{m,MgO}}$ on the phase diagram, it is optimal to compare with the data of (Mg,Fe)O melting at lowest available pressure (i.e., 3 GPa). $\Delta H_{\text{m,MgO}}$ at 3 GPa should be reasonably close to $\Delta H_{\text{m,MgO}}$ at 1 bar, which is rigorously constrained. For simplicity, we adopt the 1 bar value of $\Delta H_{\text{m,MgO}}$. Following this analysis, the calculated phase diagram at 3 GPa together with the temperature-corrected experimental data by Du and Lee[2] are consistent (Supplementary

Fig. 2).

Supplementary Note 3: Comparison with linearly extrapolated solidus melting temperatures of ferropericlase

As pointed out in the main text, the solidus temperatures of ferropericlase that we measure are much smaller than those extrapolated by a linear reduction of melting curves of pure MgO and FeO by up to ~3000 K. We plot the temperature differences in Supplementary Fig. 3 for $(Mg_{0.75}Fe_{0.25})O$, $(Mg_{0.8}Fe_{0.2})O$, $(Mg_{0.85}Fe_{0.15})O$ and $(Mg_{0.9}Fe_{0.1})O$.

Supplementary Note 4: Interpretation of local maxima in melting temperatures

In order to interpret the melting temperature variations in ferropericlase, we look to the spin transition of Fe^{2+} in (Mg,Fe)O. While it is unknown how the spin transition in iron affects the melting of (Mg,Fe)O, the spin transition has been found to influence many physical, chemical and transport properties including density[21], elastic moduli[21], element partitioning[22] and thermal/electrical conductivities[23, 24]. Nevertheless, we can make a qualitative estimate based on Lindemann's law[25]. Lindemann's law provides a simple relationship between the melting temperature and thermo-elastic properties of materials, $T_m \propto C/\rho$, where C is some combination of elastic moduli and ρ is the density [26, 27]. Both experiments and first principles computations have shown that the spin transition softens and densifies (Mg,Fe)O[28, 29]. Therefore, we expect that the spin transition tends to lower the melting temperatures based on Lindemann's law. We take $(Mg_{0.9}Fe_{0.10})O$ as an example and use the bulk modulus as the elastic constant of interest for simplicity which has been shown to decrease over a broad range (40 – 70 GPa) due to

the spin transition and reach a local minimum at ~50 GPa[28]. The local minimum bulk modulus is ~220 GPa compared with ~360 GPa if there were no spin transition. Additionally, the spin transition also increases the density of ferropericlase by ~2.4% in this pressure range[28]. As such, an overall decrease in T_m will occur between 40 – 70 GPa with the decrease peaking at ~50 GPa. After the mid-point of the spin crossover, the moduli will monotonically increase at values greater than those in the high-spin state, thus setting up a local maximum in the melting curves[28, 29]. For Earth-relevant compositions, this local maximum in T_m occurs at ~40 GPa based on the spin-state crossover range[3]. Note that the above spin-state crossover pressure range is experimentally obtained at 300 K and so high temperatures may additionally influence this analysis[30, 31, 32].

Previous ambient temperature experimental studies suggest that the elasticity of (Mg,Fe)O with Mg# between 60-94 is affected by the spin transition between 35–70 GPa and the pressures corresponding to the local minima of elastic constants are likely to increase with Fe content[3]. Assuming 1) the solidus melting temperatures at 40 GPa is only marginally affected by the spin transition compared with those at 50 GPa and 2) the increment of solidus melting temperatures of (Mg,Fe)O from 40 to 50 GPa without the effects of spin transition is a linear combination between the increments of melting temperatures of endmembers (ΔT = 70 – 280 K for Mg# 0-100), we can discriminate the amount of temperatures depressed due to spin crossover (Supplementary Fig. 4). It is clear from Supplementary Fig. 4 that the depression in solidus melting temperatures for (Mg,Fe)O with Mg# between 52 to 98 cannot merely be explained by the ±10% measurement uncertainty in the solidus melting temperature at 50 GPa. As mentioned

above, the bulk moduli of (Mg,Fe)O with those compositions are depressed by the spin transition at pressures between 35 and 70 GPa and local minimal of this depression likely located at pressures > 40 GPa[3, 33], suggesting that the extra depression in the solidus melting temperatures at 50 GPa is due to the spin transition.

To sum up, we demonstrate that the spin transition of Fe^{2+} in (Mg,Fe)O with an Mg# between 60 - 94 can at least qualitatively account for the local maximum in melting temperatures at ~40 GPa observed in this study. As the spin transition of Fe^{2+} in (Mg,Fe)O is a composition-sensitive phenomenon, pressures corresponding to the peak melting temperatures should vary with composition, which are not be observed in this study because of the coarse pressure steps.

Supplementary Note 5: Volume change upon the melting of ferropericlase

According to the Clausius–Clapeyron relation, $dT_{\mathrm{m}}/dP = \Delta V/\Delta S$, a negative melting slope (dT_{m}/dP) means that the ratio of molar volume change ΔV and molar entropy change ΔS upon melting is negative. As ΔS is generally thought to be positive during melting, our melting curves (Fig. 3 in the main text) indicate that the volume change is small and even negative during the transition of (Mg_xFe_{1-x})O (x = ~0.52-0.98) from solid to liquid at a pressure between 40 GPa to 50 GPa. Note that the specific pressure range for this negative volume change is unknown because of the lack of fine pressure steps between 40 to 50 GPa. Nevertheless, this pressure range is almost coincident with the pressures at which over half of the Fe in liquid (Mg,Fe)O become stable in the low-spin state based on a recent first-principles molecular dynamics (FPMD) simulations study[30], which indicates that the negative volume change observed in this study might be due to the spin transition of Fe in liquid (Mg,Fe)O. Since the negative

volume change only occurs between 40 and 50 GPa might imply that the population of the low-spin state Fe in liquid (Mg,Fe)O surpasses that in solid (Mg,Fe)O at the same P, T conditions. In other words, the initiation of the spin transition of Fe in solid (Mg,Fe)O might be more sluggish. This is consistent with ref. 30, which shows that at ~3000 K and 50 GPa, the fraction of low-spin state Fe in liquid ($Mg_{0.75}Fe_{0.25}$)O is ~70% (3000 K is out of the temperature range considered in ref. 30 and 70% is an estimate based on their Fig 2), larger than that in ($Mg_{0.75}Fe_{0.25}$)O solid, which is ~40% (see their Fig. 2). Indeed, at 3000 K low-spin state ($Mg_{0.75}Fe_{0.25}$)O liquid is shown to become as dense as than high-spin state ($Mg_{0.75}Fe_{0.25}$)O solid although at a pressure (90 GPa) [30] higher than what we observed in our experiments (40–50 GPa). Additionally, the possible stronger softening of the adiabatic bulk modulus of (Mg,Fe)O liquid than that observed and predicted in solid (Mg,Fe)O might partly account for the large volume reduction upon melting[34, 35].

However, in contrast to the spin transition pressures and positive Clapeyron slopes for the spin crossovers reported by ref. 30, another FPMD simulations study[31] predict ~20 to 30 GPa higher spin crossover pressure for iron in liquid ($Mg_{0.75}Fe_{0.25}$)O with a negative Clapeyron slopes for the spin transition. This discrepancy in transition pressure has been attributed to the use of the Hubbard *U* term and the sharp differences in Clapeyron slopes has mainly been attributed to entropy factors[30]. Nevertheless, the lack of knowledge about Hubbard *U* and entropy of liquid (Mg,Fe)O at high pressures and temperatures precludes the assessment of the conflicting results given by different studies. In addition to the Hubbard *U* and entropy, other simplifications made in FPMD simulations[31] such as ideal mixing between FeO and MgO liquid end-members may also introduce some uncertainty in those computational results. To conclude, the negative

volume variation of (Mg,Fe)O during melting between 40 to 50 GPa observed in this study may be the result of the spin transition of Fe in liquid (Mg,Fe)O. More studies are needed to fully understand the spin crossover of Fe in the (Mg,Fe)O liquid and the associated thermoelastic variation. More discussion concerning this topic will be included in a coming paper.

Supplementary Note 6: Pressure medium

The purpose of not using any pressure media for Mg-rich samples is to minimize the possible contamination introduced by the pressure medium. In addition, for Mg-rich samples, even if a pressure medium is used, large temperature gradients still exists due to the small absorption coefficient of the materials to laser radiation. This means that the laser can easily penetrate through the Mg-rich (Mg,Fe)O and melting would still begin from the interior of the samples, setting up temperature gradients.

For the Fe-rich samples, we use MgO as insulation and a pressure media so that we are able to heat, without which, laser heating would be difficult. We find diffusion between the MgO and Fe-rich ferropericlase and therefore do need to take this into account when applying the temperature correction due to wavelength-dependent absorption. Fortunately, in terms of contamination, MgO is fine since it is already part of the binary system we are investigating. Using a noble gas such as argon, for example, may cause lowered melting temperatures due to incorporation in to melts of this composition[2]. Additionally, due to the high temperatures anticipated for ferropericlase melting, we avoided alkali halides due to strong changes in the optical properties, which cause a rapid increase in temperature near their melting points[36].

Although there exists large temperature gradients within the sample during the laser heating due to the absence pressure media for some experiments, the correction of the effects of temperature gradients on temperature deviation is already incorporated in our inverse modeling method (See Temperature determination in main text). To summarize the consideration in Deng *et al* [1], the steady heat flow equation is solved with proper boundaries conditions to obtain the 1D axial temperature gradient. More rigorous temperature profiles can be calculated using the TempDAC code[37] with the knowledge of thermodynamic properties of materials at corresponding conditions. But unfortunately, those parameters are poorly constrained for most Earth materials at elevated pressures and temperatures. Nevertheless, the fine structure of the temperature profile obtained by rigorous thermodynamic simulation is not expected to change the axial temperature distribution within the melt much while it does alter the fine structure of the temperature profile of the solid part. As such, temperature correction will not be influenced largely by the rigorous temperature profile calculation since the hottest part (melt) dominates the effects of the temperature.

Supplementary References

1. Deng J, Du Z, Benedetti LR, Lee KKM. The influence of wavelength-dependent absorption and temperature gradients on temperature determination in laser-heated diamond-anvil cells. *J Appl Phys* **121**, (2017).

2. Du Z, Lee KKM. High-pressure melting of MgO from (Mg,Fe)O solid solutions. *Geophysical Research Letters* **41**, 8061-8066 (2014).

3. Glazyrin K, Miyajima N, Smith JS, Lee KKM. Compression of a multiphase mantle assemblage: Effects of undesirable stress and stress annealing on the iron spin state crossover in ferropericlase. *Journal of Geophysical Research: Solid Earth* **121**, 2015JB012321 (2016).

4. Tsuchiya T, Wentzcovitch RM, da Silva CRS, de Gironcoli S. Spin transition in magnesiowustite in earth's lower mantle. *Phys Rev Lett* **96**, (2006).

5. Lin J-F*, et al*. Spin Transition Zone in Earth's Lower Mantle. *Science* **317**, 1740-1743 (2007).

6. Van Orman JA, Fei YW, Hauri EH, Wang JH. Diffusion in MgO at high pressures: Constraints on deformation mechanisms and chemical transport at the core-mantle boundary. *Geophysical Research Letters* **30**, (2003).

7. Ito Y, Toriumi M. Pressure effect of self-diffusion in periclase (MgO) by molecular dynamics. *Journal of Geophysical Research: Solid Earth* **112**, B04206 (2007).

8. Ita J, Cohen RE. Effects of Pressure on Diffusion and Vacancy Formation in MgO from Nonempirical Free-Energy Integrations. *Phys Rev Lett* **79**, 3198-3201 (1997).

9. Cohen RE, Weitz JS. The Melting Curve and Premelting of MgO. In: *Properties of Earth and Planetary Materials at High Pressure and Temperature* (ed^(eds). American Geophysical Union (1998).

10. Yamazaki D, Irifune T. Fe-Mg interdiffusion in magnesiowustite up to 35 GPa. *Earth and Planetary Science Letters* **216**, 301-311 (2003).

11. Mackwell S, Bystricky M, Sproni C. Fe–Mg Interdiffusion in (Mg,Fe)O. *Physics and Chemistry of Minerals* **32**, 418-425 (2005).

12. Holzapfel C, Rubie DC, Mackwell S, Frost DJ. Effect of pressure on Fe–Mg interdiffusion in (FexMg1−x)O, ferropericlase. *Physics of the Earth and Planetary Interiors* **139**, 21-34 (2003).

13. Zhang L, Fei YW. Melting behavior of (Mg,Fe)O solid solutions at high pressure. *Geophysical Research Letters* **35**, (2008).

14. Fischer RA, Campbell AJ. High-pressure melting of wüstite. *American Mineralogist* **95**, 1473-1477 (2010).

15. Vočadlo L, Price GD. The melting of MgO — computer calculations via molecular dynamics. *Physics and Chemistry of Minerals* **23**, 42-49 (1996).

16. Alfe D. Melting curve of MgO from first-principles simulations. *Phys Rev Lett* **94**, (2005).

17. Frost DJ, *et al*. Partitioning of oxygen between the Earth's mantle and core. *Journal of Geophysical Research: Solid Earth* **115**, B02202 (2010).

18. Komabayashi T. Thermodynamics of melting relations in the system Fe-FeO at high pressure: Implications for oxygen in the Earth's core. *Journal of Geophysical Research: Solid Earth* **119**, 4164-4177 (2014).

19. Frost DJ. Fe2+-Mg partitioning between garnet, magnesiowustite, and (Mg,Fe)(2)SiO4 phases of the transition zone. *American Mineralogist* **88**, 387-397 (2003).

20. JANAF. *Thermochemical Tables* 2nd edn. NSRDS- NBS 37 (1971).

21. Badro J. Spin Transitions in Mantle Minerals. *Annual Review of Earth and Planetary Sciences, Vol 42* **42**, 231-248 (2014).

22. Badro J, *et al*. Iron partitioning in Earth's mantle: Toward a deep lower mantle discontinuity. *Science* **300**, 789-791 (2003).

23. Ohta K, Hirose K, Onoda S, Shimizu K. The effect of iron spin transition on electrical conductivity of (Mg,Fe)O magnesiowüstite. *Proceedings of the Japan Academy Series B, Physical and Biological Sciences* **83**, 97-100 (2007).

24. Goncharov AF, Struzhkin VV, Jacobsen SD. Reduced Radiative Conductivity of Low-Spin (Mg,Fe)O in the Lower Mantle. *Science* **312**, 1205-1208 (2006).

25. Lindemann FA. über die Berechnung Molecular Eigenfrequnzen. *Physikalische Zeitschrift* **11**, 609-612 (1910).

26. Karato SI. Effects of pressure and water. *Deformation of Earth Materials: An Introduction to the Rheology of Solid Earth*, 168-198 (2008).

27. Yamazaki D, Karato S. Some mineral physics constraints on the rheology and geothermal structure of Earth's lower mantle. *American Mineralogist* **86**, 385-391 (2001).

28. Marquardt H, Speziale S, Reichmann HJ, Frost DJ, Schilling FR. Single-crystal elasticity of (Mg0.9Fe0.1)O to 81 GPa. *Earth and Planetary Science Letters* **287**, 345-352 (2009).

29. Wu Z, Justo JF, Wentzcovitch RM. Elastic Anomalies in a Spin-Crossover System: Ferropericlase at Lower Mantle Conditions. *Phys Rev Lett* **110**, 228501 (2013).

30. Ghosh DB, Karki BB. Solid-liquid density and spin crossovers in (Mg, Fe)O system at deep mantle conditions. *Sci Rep-Uk* **6**, 37269 (2016).

31. Holmstrom E, Stixrude L. Spin crossover in liquid (Mg,Fe)O at extreme conditions. *Phys Rev B* **93**, (2016).

32. Wentzcovitch RM, Justo JF, Wu Z, da Silva CRS, Yuen DA, Kohlstedt D. Anomalous compressibility of ferropericlase throughout the iron spin cross-over. *Proceedings of the National Academy of Sciences* **106**, 8447-8452 (2009).

33. Crowhurst JC, Brown JM, Goncharov AF, Jacobsen SD. Elasticity of (Mg,Fe)O Through the Spin Transition of Iron in the Lower Mantle. *Science* **319**, 451-453 (2008).

34. Wu Z, Justo JF, da Silva CRS, de Gironcoli S, Wentzcovitch RM. Anomalous thermodynamic properties in ferropericlase throughout its spin crossover (vol 80, 014409, 2009). *Phys Rev B* **80**, (2009).

35. Fei Y, *et al*. Spin transition and equations of state of (Mg, Fe)O solid solutions. *Geophysical Research Letters* **34**, L17307 (2007).

36. Boehler R, Ross M, Boercker DB. High-pressure melting curves of alkali halides. *Phys Rev B* **53**, 556-563 (1996).

37. Rainey ESG, Hernlund JW, Kavner A. Temperature distributions in the laser-heated diamond anvil cell from 3-D numerical modeling. *J Appl Phys* **114**, 204905 (2013).
